# Association between Actual and Perceived Obesity Weaker among Black than White Children

**DOI:** 10.3390/bs8050048

**Published:** 2018-05-14

**Authors:** Maryam Moghani Lankarani, Shervin Assari

**Affiliations:** 1 Department of Psychiatry, University of Michigan School of Medicine, Ann Arbor, MI 48104, USA; lankaranii@yahoo.com; 2 Center for Research on Ethnicity, Culture and Health, School of Public Health, University of Michigan, Ann Arbor, MI 48104, USA

**Keywords:** obesity, overweight, perceived overweight, weight control, Blacks, race, gender

## Abstract

Although actual obesity is expected to be associated with perceived overweight, some recent studies in adults have suggested that this link may be smaller for Blacks than Whites. It is unknown, however, whether the same trend holds for children or not. This study explored the heterogeneity of the association between actual and perceived obesity in a national sample of American children by race, gender, and their intersection. Health Behavior in School-Aged Children (HBSC), 2009–2010, is a national study of children 17 years or less in the United States. This analysis included a total number of 8860 children, including 6581 (74.28%) White and 2279 (25.72%) Black children. Actual obesity, defined as a body mass index (BMI) greater than 95% of the age- and gender-percentile, was the independent variable. Perceived overweight was the main outcome. We ran linear regression models with and without interaction terms between race, gender, and actual obesity. We also ran race- and gender-specific linear regression models. In the pooled sample, actual obesity was positively associated with perceived overweight. We found an interaction between race and obesity, suggesting stronger association between actual obesity and perceived overweight for White than Black children. Gender or intersection of race and gender did not alter the association between actual obesity and perceived overweight. The link between actual obesity and perceived overweight depends on race of the child. Inaccurately perceived weight may be one of many mechanisms behind the disproportionately higher rate of obesity burden among Black children in the United States. As perceived overweight plays a salient role for weight control behaviors, Black children with obesity may need some help to perceive themselves as obese. Training programs should target Blacks to increase the accuracy of their weight and body size evaluation and perception as an essential step for reducing the burden of obesity among Black children.

## 1. Introduction

With over 60% of Americans being overweight or obese [1,2,3,4], the obesity epidemic is a pressing public health concern in the United States (U.S.), affecting both American children and adults. All-cause mortality rate increases even with a moderate excess in weight [1]. Obesity increases risk of metabolic disorders, hypertension, stroke, cardiovascular disease, and cancers [2]. In the U.S., with 300,000 annual deaths, obesity is second only to tobacco as a leading cause of death [5]. Given the increasing trend of prevalence of obesity [4], obesity-related mortality and morbidity is projected to increase in the near future [6]. As behaviors and cognitive elements play a role in the increasing trend in obesity [2,3], there is a need to better understand the cognitive elements associated with obesity. The results of such epidemiological studies have public health implications and can inform policies, programs, and practices that may reduce the burden of obesity [2,7,8].

Perceiving oneself as overweight is essential for individuals with obesity to initiate weight control behaviors and the desire to lose weight [8]. As a result, increasing accuracy of one’s own perception regarding overweight status is a desired outcome for healthy weight programs [9]. Information on the heterogeneity of the link between actual and perceived overweight has the potential to explain why some populations have a lower tendency to participate in weigh control programs or engage in weight reduction behaviors (e.g., exercise and diet) [10]. Such information may have implications for enhancement of populations’ participation in weight control programs, particularly for minorities with less an accurate perception and evaluation of their obesity, which puts them at a disproportionately high risk for lack of participation in weight loss programs [11,12]. To better understand the association between actual obesity and related cognitive elements (e.g., perceived overweight, weight loss intentions, and weight loss behaviors) [11,13], more research is needed on how race, gender, and the intersection of these variables alters these associations.

### Aim

The current study tested the heterogeneity in the association between actual obesity and perceived overweight in a national sample of children based on race, gender, and their intersection. We hypothesized a weaker association among Black children than among White children.

## 2. Materials and Methods

### 2.1. Design and Setting

The Health Behavior in School-Aged Children (HBSC) is a cross-national, school-based study, which began in 1982 to gather health and health behaviors in children and adolescents [14]. In collaboration with the World Health Organization (WHO) Regional Office in Europe, the HBSC is conducted in U.S. and 29 other participating countries. The study follows up every four years since the 1985–1986 school year. The HBSC is a school-based survey. The HBSC’s target population is school-aged children between 10 and 17 years of age. This age group represents early and middle adolescence. The HBSC uses a nationally representative sample in each participating country including the U.S. The total sample consists of approximately 1500 adolescents from each age group. The United States, however, was chosen to implement the HBSC survey outside of the four-year cycle. The HBSC measures a wide range of factors such as demographics, socioeconomics, family relations, health attitudes, health behaviors (diet and substance use), and prosocial and antisocial behaviors. More information on the HBSC methodology is available elsewhere [15].

### 2.2. Ethics

The HBSC was approved by the National Institute of Child Health and Human Development (NICHD) Institutional Review Board (IRB). The study obtained assent from the participating children and written consent from parents. The HBSC is funded by the NICHD.

### 2.3. Sample and Participants

The HBSC study obtained a nationally-representative sample of American children. For that purpose, the HBSC study used a three-stage stratified sampling design. The HBSC oversampled African-Americans/Blacks and Hispanics. The three stages of the sampling included (1) school districts; (2) schools; and (3) school classes. Sampling included nine strata of census regions, and three strata of grades within each census region. As a result, there is a sampling weight that should be taken into account when analyzing the HBSC data.

### 2.4. Data Collection

Data were collected using anonymous self-administered questionnaires. Questionnaires were distributed in grades 6–10. Of the 327 schools included in the HBSC study, 230 (70.3%) had students who were 11, 13, or 15 years old. A total of 10607 students were eligible for inclusion in the current study, of whom 85% had responded to the HBSC survey. Eligible schools had students in at least one of grades 6–10. Considerations for eligibility of schools include students 11, 13, and 15 years old; school type; geographic location; and ethnic composition of the school (as the study over-sampled Black students) [14]. From all the eligible students (who were either 11, 13, and 15 years old), 85% (9016) of students participated in the HBSC study.

### 2.5. Measures

Socio-demographic factors: The study measured race, ethnicity, age, gender, and socioeconomic status (SES). Gender was measured as male (referent group) or female. Race was measured as self-identified, and included two categories: Caucasian/Whites (referent group) and African American/Blacks. Ethnicity was also self-identified as non-Hispanic (referent group) versus Hispanic. As race and ethnicity were measured and treated as independent variables, White and Black in this study do not mean non-Hispanic White or non-Hispanic Black. As there was insufficient sample size for other racial groups, we did not include other minorities. The study measured SES indicators such as subjective SES and maternal employment. Maternal employment was a dichotomous variable measured as unemployed (referent group) versus employed. Subjective SES included (1) very well off; (2) quite well off; (3) average; (4) not very well off; and (5) not at all well off. This variable was treated as a continuous measure.

Obesity: The study defined childhood obesity based on the comparison of body mass index (BMI) with the 95th percentile BMI of the age- and gender-specific norms. BMI scores were calculated based on self-reported height and weight. The variable obesity was treated as a dichotomous variable with the following levels: obese (those with BMI % equal or greater than 95th percentile) versus non-obese (those with BMI % less than 95, (referent category)) [16,17]. Although there is a tendency for underestimation of self-reported weight and an overestimation of self-reported height [18,19] BMI based on self-reported height and weight is strongly associated (kappa 0.73) with BMI based on direct measurement [20].

Perceived overweight: Perceived overweight was measured using a single item measure. The item read: “Do you consider yourself (1) much too thin; (2) a bit too thin; (3) about the right size; (4) a bit too fat; or (5) much too fat?”. Scores had a potential range of 1 to 5, with a higher score indicating higher perceived overweight. This variable was treated as a continuous measure.

### 2.6. Statistical Analysis

We used Stata 13.0 for data analysis, using sub-population survey commands. To accommodate the study weights, we re-estimated our SES using Taylor series linearization. All means and proportions (frequencies) with their 95% confidence intervals (CIs) apply sampling weights. Survey linear regression models were used for multivariable analysis, by considering perceived overweight as the dependent variable, actual obesity as the independent variable, and age and family SES as covariates. Race and gender were focal moderators. We ran models in the pooled sample and also based on race and gender. Adjusted regression coefficients (*B*) and 95% CIs were reported. All the *p*-values less than 0.05 were considered statistically significant.

## 3. Results

### 3.1. Descriptive Statistics

Table 1 summarizes the results of descriptive statistics. Children were 13.1 years old on average and 48% were female. From all the participants, 18.5% were Black and 6.2% were Hispanic. About 12.6% of the participants were obese. Most participants found themselves about the right size, and 27.5% reported themselves as overweight. From all participants, 76% had an employed mother and 59.4% had both parents in the household.

### 3.2. Linear Regressions in the Pooled Sample

Table 2 summarizes the results of four linear regressions in the pooled sample. Model 1 only tested the main effects. Model 2 also included two interaction terms: race × obesity and gender × obesity. Model 3 also included another interaction term: race × gender. Model 4 also included another interaction terms: race × gender × obesity.

Based on Model 1, in the pooled sample, actual obesity was positively associated with perceived overweight. This model also showed that Blacks have a higher risk of perceiving themselves as overweight (*B* = 0.16, 95% CI = 0.12–0.20). However, Hispanics were less likely to perceive themselves as overweight (*B* = −10.12, 95% CI = −0.17–−0.07). Lower subjective SES also increased risk of perceiving themselves as overweight (*B* = 0.03, 95% CI = 0.00–0.05).

Model 2 found an interaction between race and obesity (*B* = −0.15, 95% CI = −0.29–−0.02), suggesting stronger association between actual obesity and perceived overweight for White than Black children. Based on Models 2–4, gender or intersection of race and gender did not alter the association between actual obesity and perceived overweight (*p* > 0.05 for all interactions). (Table 2).

### 3.3. Linear Regressions by Race and Gender

Table 3 summarizes the results of linear regressions by race and gender. Although actual obesity was positively associated with perceived overweight in Whites and Blacks, the magnitude of the association was larger for Whites (*B* = 0.82) than for Blacks (*B* = 0.65). (Table 3).

## 4. Discussion

The findings of this study suggest that although actual obesity and perceived overweight are closely linked, this association is weaker for Black, compared to White, children. The association between actual obesity and perceived overweight seems not to depend on gender or the intersection of race and gender.

Factors associated with risk of obesity in Black families are multilevel. Blacks and Hispanics are at higher risks of having low SES, hunger, and food insecurity, as well as of poor health behaviors relating to exercise and healthy diets. Black and Hispanic communities are also more likely to be located in food deserts that affect diet quality, and with less access to safe places that promote physically active life styles. High crime rates of poor neighborhoods also contribute to the risk of obesity. Black communities have more fast food restaurants and lower availability of nutritious foods. All these factors contribute to the higher burden of obesity in Black communities [1,2]. This study also suggests that inaccuracy in their perceived weight may also be another reason for such disparities.

The findings reported here are compatible with two previous studies in adult samples [21,22]. Both studies used data from the National Survey of American Life (NSAL), a nationally representative study of American adults. In the first study, the association between overweight status and intention to lose weight was weaker for Blacks than Whites and for men than women [22]. In the second study, perceived overweight fully mediated the effect of actual obesity on intention for weight control among White women and Black men, but not White men and Black women [21].

These studies collectively suggest that the links between actual weight, weight perception, and weight loss intention vary by race, with weaker associations for Blacks than Whites. These studies show that Blacks with obesity are at a disadvantage for accurate perceptions of their body weight and the development of intentions for weight loss compared to Whites. These racial variations may at least in part explain racial disparities in obesity burden [4]. This finding may also help explain why obesity is associated with smaller psychosocial costs in Blacks than in Whites [15,23,24,25,26,27,28]. For instance, Blacks with obesity are less likely to be depressed than Whites with obesity [15,24,25,26,27,28,29,30]. Similar findings are shown in youth [27,28], adults [24,26], and older adults [25]. We argue that one reason behind smaller psychosocial costs of obesity in Blacks is their weaker association between actual obesity and perceived overweight.

### 4.1. Implications

The current findings have clinical and public health implications. Interventions that operate based on perceived overweight and motivation to lose weight may require an extra boost for Blacks compared to Whites. With this argument, healthy weight programs in the U.S. may benefit from tailoring their approaches based on race [18,21].

Our findings help us understand the central and contextual role of race as a salient moderator on the above linkages. Our findings also provide an explanation for some of the racial differences reported in the literature on the above topics. We argue that universal interventions that ignore sub-group variations in the links between actual weight, perceived weight, desired weight, and weight control behaviors may not generate the maximum efficacy in encouraging individuals with obesity to reduce weight. Additional programs are needed for Black children to increase their readiness to develop intention for weight control, which is a core element of weight loss interventions [31]. Findings are also relevant to the efficacy of public health programs that wish to reduce risks of cardio-metabolic conditions such as obesity in Blacks [32,33,34,35,36,37,38,39,40,41,42].

### 4.2. Limitations

The study is not free from limitations. Our first limitation is possible measurement bias. We measured actual obesity using self-reported data, and perceived overweight using a single-item measure. Single-item measures are not ideal to measure such psychological and cognitive constructs. However, perceived overweight has been commonly measured using single-item measures. We also did not measure some confounders or behaviors such as diet, exercise, food insecurity, body size dissatisfaction, and intention to reduce weight. Other factors such as body image dissatisfaction, perceived social pressure related to thinness, and other weight related factors should also be considered [43,44]. Some research has shown that attitudes of the parents have a role in these differences. Black parents tend to perceive their children to have healthy weight when they are actually over weight. More research is needed on how parents’ attitudes and weight perception would jointly shape the child’s view of their weight. Actual weight should be measured not using self-reported but measured weight and height. More research is needed on how increasing overweight awareness would enhance efficacy of weight management programs. It is also unknown whether such enhancement of accuracy of body image will differently increase weight loss across racial groups.

### 4.3. Future Research

Research is still needed on the intersections of race, ethnicity, gender, place, class, and age, on the complex processes that are involved in maintaining healthy weight. More research is needed on the non-linear links between actual weight, perceived weight, desired weight, accepted weight, body image, body satisfaction, weight loss intention, and weight control behaviors across population groups [45,46,47,48,49]. Social groups may vary in their desirable weight, which may influence their perception of own-weight and intention to lose weight. Research shows that location of residence, in terms of region of the country, impacts cultural preferences in body weight. The effects of location of residency on the association between actual and perceived body weight, and differences in this association between Black and White children should be investigated. Future research is needed to replicate these findings using multi-item measures of perceived overweight and eating behaviors. Future research should use a longitudinal design, with multiple observations over time.

## 5. Conclusions

To summarize, we found that actual obesity and perceived overweight were more closely associated in Whites than Blacks. The degree of salience of perceived own weight in the cognitive processes that result in weight loss behaviors may be different for Black and White children, suggesting that Black children with obesity may require some tailored programs to increase their sensitivity to their weight and body size. These are important, as decisions about weight loss may require the individual to evaluate themselves as obese. Perceived overweight may be a key element of obesity treatment for children, particularly Black children. Weight control programs may consider such differences between Black and White individuals with obesity.

## Figures and Tables

**Table 1:** Descriptive of demographics, socioeconomics, actual obesity, and perceived overweight.

Characteristic		
	Mean	95% CI
Age	13.09	13.03–13.14
Subjective socioeconomic status (SES)	2.54	2.51–2.57
Perceived overweight	3.17	3.15–3.19
	%	**95% CI**
Race		
White	81.52	80.29–82.69
Black	18.48	17.31–19.71
Ethnicity		
Non-Hispanic	93.77	93.02–94.44
Hispanic	6.23	5.56–6.98
Gender		
Male	51.60	49.99–53.22
Female	48.40	46.78–50.01
Actual obesity		
No	87.41	86.33–88.42
Yes	12.59	11.58–13.67
Perceived weight		
Much too thin	1.33	1.03–1.72
A bit too thin	9.99	9.10–10.96
About the right size	61.22	59.64–62.77
A bit too fat	24.84	23.49–26.24
Much too fat	2.62	2.13–3.21
Employed mother		
No	23.96	22.54–25.43
Yes	76.04	74.57–77.46
Both parents in the household		
No	40.58	39.00–42.17
Yes	59.42	57.83–61.00
Subjective socioeconomic status (SES)		
Very well off	17.49	16.19–18.87
Quite well off	25.09	23.75–26.48
Average	47.20	45.59–48.81
Not very well off	8.24	7.42–9.14
Not at all well off	1.98	1.58–2.49

Source: Health Behavior in School-Aged Children (HBSC), 2009–2010 [14]. Sample size: 8860 children including 6581 (74.28%) White and 2279 (25.72%) Black children. CI: confidence interval.

**Table 2:** Association between actual obesity and perceived overweight among children in the pooled sample

	Model 1		Model 2		Model 3		Model 4	
	*B*	95% CI	*B*	95% CI	*B*	95% CI	*B*	95% CI
Actual obesity	0.79 ***	0.73–0.85	0.86 ***	0.77–0.94	0.86 ***	0.78–0.94	0.87 ***	0.78–0.96
Ethnicity (Hispanic)	−0.12 ***	−0.17–−0.07	−0.10 ***	−0.15–−0.04	−0.13 ***	−0.21–−0.06	−0.13 **	−0.20–−0.05
Gender (Female)	−0.01	−0.08–0.07	−0.01	−0.08–0.07	−0.01	−0.08–0.07	−0.01	−0.08–0.07
Race (Black)	0.16 ***	0.12–0.20	0.09	−0.02–0.21	0.14 *	0.01–0.28	0.11	−0.06–0.28
Age	0.01	0.00–0.02	0.01	0.00–0.02	0.01	0.00–0.02	0.01	0.00–0.02
Mother employed	−0.04	−0.09–0.01	−0.04	−0.09–0.01	−0.04	−0.09–0.01	−0.04	−0.09–0.01
Both parents present in the family	−0.03	−0.07–0.02	−0.02	−0.07–0.02	−0.02	−0.07–0.02	−0.02	−0.07–0.02
Subjective socioeconomic status (SES) (low)	0.03 *	0.00–0.05	0.03 *	0.00–0.05	0.03 *	0.00–0.05	0.03 *	0.00–0.05
Black × Obesity	-	-	−0.15 *	−0.29–−0.02	−0.15 *	−0.29–−0.02	−0.19 *	−0.36–−0.02
Female × Obesity	-	-	0.08	−0.05–0.20	0.08	−0.04–0.20	0.10	−0.04–0.25
Black × Female	-	-	-	-	−0.07	−0.17–0.03	−0.06	−0.16–0.05
Black × Female × Obesity	-	-	-	-	-	-	0.08	−0.19–0.36
Intercept	2.86 ***	2.67–3.06	2.86 ***	2.66–3.06	2.86 ***	2.66–3.06	2.86 ***	2.66–3.06

Outcome: Perceived overweight. Source: Health Behavior in School-Aged Children (HBSC), 2009–2010 [14]. Sample size: 8860 children including 6581 (74.28%) White and 2279 (25.72%) Black children. *B*: adjusted regression coefficient, CI: confidence interval; *p < 0.05 **p < 0.001, *** *p* < 0.01.

**Table 3:** Association between actual obesity and perceived overweight among children by race and gender

	Model 1		Model 2		Model 3		Model 4	
	*B*	95% CI	*B*	95% CI	*B*	95% CI	*B*	95% CI
	Whites		Blacks		Males		Females	
Actual obesity	0.82 ***	0.75–0.89	0.65 ***	0.54–0.77	0.82 ***	0.74–0.90	0.74 ***	0.64–0.83
Ethnicity (Hispanic)	0.00	−0.09–0.09	−0.01	−0.17–0.14	−0.15 ***	−0.22–−0.08	−0.08 ***	−0.15–−0.01
Gender (Female)	0.15 ***	0.10–0.19	0.21 ***	0.12–0.30	-	-	-	-
Race (Black)	-	-	-	-	0.01	−0.10–0.12	−0.03	−0.13–0.07
Age	0.02 **	0.00–0.03	−0.02	−0.05–0.01	−0.01	−0.03–0.01	0.04 ***	0.02–0.05
Mother employed	−0.04	−0.09–0.02	−0.05	−0.14–0.04	−0.03	−0.11–0.04	−0.04	−0.11–0.02
Both parents present in the family	−0.03	−0.09–0.02	0.03	−0.05–0.12	−0.03	−0.09–0.04	−0.02	−0.08–0.04
Subjective socioeconomic status (SES) (low)	0.04 **	0.01–0.06	−0.01	−0.06–0.04	0.01	−0.02–0.05	0.04 ***	0.01–0.07
Intercept	2.74 ***	2.51–2.97	3.25 ***	2.88–3.61	3.21 ***	2.91–3.51	2.64 ***	2.40–2.87

Outcome: Perceived overweight. Source: Health Behavior in School-Aged Children (HBSC), 2009–2010 [14]. Sample size: 8860 children including 6581 (74.28%) White and 2279 (25.72%) Black children, *B*: adjusted regression coefficient, CI: confidence interval, **p < 0.001, *** *p* < 0.01.
